# Molecular Mechanism of Yisui Shengxue Granule, a Complex Chinese Medicine, on Thalassemia Patients Suffering from Hemolysis and Anemia of Erythrocytes

**DOI:** 10.1155/2014/213782

**Published:** 2014-12-10

**Authors:** Na-Li Chu, Zhi-kui Wu, Xin-Hua Zhang, Su-Ping Fang, Wen-Juan Wang, Yan-Ling Cheng

**Affiliations:** ^1^Guang'anmen Hospital, China Academy of Chinese Medical Sciences, Beijing 100053, China; ^2^303 Hospital of Chinese People's Liberation Army (PLA), Nanning 530000, China; ^3^The Capital Medical University, Beijing 100069, China

## Abstract

The objective of this study was to investigate the therapeutic biological mechanism of Yisui Shengxue Granule (YSSXG), a complex Chinese medicine, on the hemolysis and anemia of erythrocytes from patient with thalassemia disease. Sixteen patients with thalassemia (8 cases of *α*-thalassemia and 8 cases of *β*-thalassemia) disease were collected and treated with YSSXG for 3 months. The improvements of blood parameter demonstrated that YSSXG had a positive clinical effect on patients with thalassemia disease. For patients with *α*-thalassemia disease, RT-PCR showed that YSSXG upregulated the relative mRNA expression level of *α*-globin to *β*-globin and downregulated DNMT1, DNMT3a, and DNMT3b mRNA compared with pretreatment. Western blotting showed that YSSXG downregulated the expression of DNMT1 and DNMT3a. For patients with *β*-thalassemia disease, the relative expression level of ^A^ *γ*-globin to *α*-globin had an increasing trend and the level of BCL11A mRNA expression obviously increased. For all patients, RT-PCR showed that YSSXG upregulated mRNA expression of SPTA1 and SPTB. Activities of SOD and GSH-Px significantly increased and MDA obviously reduced on erythrocyte and blood serum after YSSXG treatment. TEM showed that YSSXG decreased the content of inclusion bodies. Activities of Na^+^K^+^-ATPtase and T-ATPtase of erythrocyte increased significantly after YSSXG treatment. This study provides the basis for mechanisms of YSSXG on thalassemia suffering with hemolysis and anemia of erythrocytes from patient.

## 1. Introduction

Thalassemia encompasses a spectrum of hereditary anemias characterized by reduced or absent production of one or more globin chains [1]. Normal human adult hemoglobin (Hb)A(HbA) consists of two pairs of globin chains, *α*2 *β*2, of which synthesis is normally tightly coordinated to ensure equal production. The molecular defect leads to an imbalance of *α*/*β*-globin chains synthesis. The excess globin chain depositing on the red cell membrane induces immune and oxidant injury, causes secondary enzymes and metabolic abnormalities, and results in the decreasing deformability and mechanical stability of RBC, which cause hemolysis and ineffective hematopoiesis [2, 3]. Transfusion is the major treatment for thalassemia, which can cause splenomegaly and hyperthyroidism and aggravate the anemia and other cells damage. Some people try to use an alkylating agent, butyrate, and its derivatives, Myleran, and other drugs to treat thalassemia disease, but these drugs have strong side effects which limit the application in clinical practice. It is difficult to popularize the therapy of bone marrow and stem cell transplantation and gene therapy which were reported about individual cases in clinical practice. Based on the “kidney essence marrow” theory, YSSXG which is a typical prescription of kidney-nourishing and marrow-replenishing therapy has made a positive effect on the treatment of two different genotypes (*α*- and *β*-type) thalassemia disease in high incidence area of Guangxi [4–7]. To verify scientific effect of kidney-nourishing and marrow-replenishing therapy on thalassemia, we collected 16 cases of thalassemia patients in Nanning, Guangxi (8 cases with *α*-thalassemia and 8 cases with *β*-thalassemia) and explored the biological mechanisms of clinical effect based on pathological mechanism of hemolysis and anemia.

## 2. Materials and Methods

### 2.1. Diagnostic Criteria

The diagnostic criteria of western medicine for intermedia thalassemia were referred to “Criteria for Diagnosis and Therapeutic Effect on Hematopathy” edited by Zhang [8].

### 2.2. Inclusion, Exclusion, and Withdrawal Criteria

The inclusion criteria were (1) for the people who accord with the diagnostic criteria of western medicine. (2) The age of patients ranged from 4 to 40 years old. (3) Patients volunteered to participate in this study and they signed informed consent forms.

The exclusion criteria were as follows: (1) the patients who have immunologic deficiency or primary diseases of the liver, kidney, or blood system, (2) the patients who are pregnant, (3) the patients who are allergic to this drug ingredients, and (4) the patients who took the antianemia drugs in the last two months.

The withdrawal criteria were as follows: (1) the patients who did not take drugs following requirements of the study protocol, quit by themselves, or were lost to follow-up and (2) patients who took any antianemia drugs and received blood transfusion in the treatment were rejected.

### 2.3. Study Population and General Data

Sixteen outpatients (8 cases of *α*-thalassemia and 8 cases of *β*-thalassemia) were enrolled from the Department of Hematology, 303 Hospital of Chinese People's Liberation Army (PLA), during the period from October to December in 2013. Sixteen patients were 7 to 26 years old, with an average of 13.19 ± 5.47 years. The patients of Zhuang and Han nationalities were 8 cases, respectively. Of the 16 patients, 10 were males and 6 were females. Cases of mild anemia were 3 and cases of moderate anemia were 13. Three genotypes of *α*-thalassemia were detected, 1 case of genotype of—^SEA^/*α* ^4.2^, 1 case of genotype of—^SEA^/*α* ^3.7^, 6 cases of genotype of—^SEA^/*α* ^CS^ *α*. Six genotypes of *β*-thalassemia were detected, 2 cases of genotypes of 17/28, 2 cases of genotypes of 41-42/-28, 1 case of genotypes of 17/E, 1 case of genotypes of 17/N, 1 case of genotypes of 28/IVS1-1, and 1 case of genotypes of 43/17.

### 2.4. Drugs and Interventions

A self-control study was carried out. The course of treatment was 3 months. YSSXG was produced by Guang'anmen Hospital Preparation Factory according to the protocol described in patent (number CN1872182, batch number 20120516). The modified YSSXG consisted of 11 Chinese herbal medicinal components: Rhizoma Kaempferiae, Radix Polygoni Multiflori, Radix Rehmanniae Preparata, Radix Astragali, Radix Codonopsis, Radix Angelicae Sinensis, Fructus Psoraleae, Colla Corii Asini, Caulis Spatholobi, Carapax Trionycis, and Fructus Amomi. A pack of granules contains 10 g powder (1 g powder contains 2.368 g crude drug).

Patients aged 2 to 6 years old were instructed to take half a pack of granules twice daily; aged 6 to 10 years old, a full pack of granules twice daily; and aged over 10 years old, a pack of granules thrice daily. The granules are dissolved in warm water and taken orally. Patients were required to have no blood transfusion during the observation period and asked to insist on the treatment regimen.

### 2.5. Blood Sample Collection

Venous blood samples (5 mL) were collected into EDTA tubes from 16 patients with thalassemia disease before and after YSSXG treatment. The blood, mixed by horizontal shaker, was centrifuged at 1700 r/min for 10 min. The upper layer of the plasma was discarded. The lower layer of blood cells which was shopped at lymphocyte separation medium were added saline to 5 mL, and then centrifuged at 2500 r/min for 20 min. Mononuclear cell layer which was located between the plasma and the lymphocyte separation liquid was drawn to 15 mL centrifuge tube, then was added saline to 10 mL and centrifuged at 1800 r/min for 10 min twice. The above mixture was discarded after centrifuging and the precipitation mononuclear cells were added to 1 mL TRIZOL reagent and stored in EP tubes frozen at −80°C refrigerator for RNA extraction. After the blood isolated by lymphocytes separation medium, red blood cells were at the lowest layer. The red blood cells were added saline to 10 mL and centrifuged at 1700 r/min for 5 min, whose supernatant was discarded. Pure red blood cells was stored at frozen pipes for −80°C refrigerator. Venous blood samples (4 mL) were collected into non-anticoagulant tube from 16 patients with thalassemia disease before and after YSSXG treatment, which were centrifuged at 1700 r/min for 10 min to obtain blood serum for detection.

### 2.6. Indicators of Observation and Detection

#### 2.6.1. Reagents

Lymphocyte separation medium was purchased from Solarbio, Co. (China) (batch number P8610). Trizol reagent was purchased from Invitrogen, USA (batch number 14105). Test kits for superoxide dismutase (SOD), malonaldehyde (MDA), and glutathione peroxidase (GSH-Px) were purchased from Nanjing Jiancheng Institute of Bioengineering (batch number 20140224). RNA Mini Kit was purchased from Tianjin, Co. (China) (batch number 139315390). RevertAid First Strand cDNA Synthesis Kit was purchased from Fermentas, LT (Lithuania) (batch number 00146314). Power SYBR Green PCR Master Mix was purchased from ABI, USA (batch number 1402445). The antibody against DNMT1 and DNMT3a was purchased from CST (batch number 5119, 2160), DNMT3b was purchased from Abcam (batch number ab79822), and *β*-actin was purchased from Beijing Zhongshan Golden Bridge Biological Technology Co. (China) (batch number TA-09). BCA Protein Assay Kit was purchased from Beijing ComWin Biotech Co. Ltd. (batch number 02912E). Test Kit for Mini ATP enzyme (Na^+^K^+^, Ca^2+^Mg^2+^, and T-ATP enzyme) of RBC was purchased from Nanjing Jiancheng Institute of Bioengineering (batch number 20140606).

#### 2.6.2. Detection of Blood Parameters

Blood parameters, Hb, RBC count, and reticulocyte percent (Ret) of all patients were measured dynamically before and after treatment by using a cell DYN 3700 automatic blood analyzer (USA). The parameter of HbF was only measured in *β*-thalassemia patients by the method of high-performance liquid chromatography (HPLC) through using Bio-Rad Variant II System (Variant, Bio-Rad, Hercules, CA, USA).

#### 2.6.3. Detection of SOD, MDA, and GSH-Px

The contents of SOD, GSH-Px, and MDA were detected by the digestive method, SOD by the xanthine oxidase method, GSH-Px by the enzyme to catalyze the reaction of hydrogen peroxide (H_2_O_2_) and GSH using 5,5′-dithiobis(2-nitrobenzoic acid, DTNB) to determine the quantity of remainder GSH, and MDA by thiobarbituric acid method.

The detection steps of erythrocyte SOD were as follows. (1) 20 *μ*L of erythrocyte sedimentation was added to 200 *μ*L deionized water and fully mixed. (2) The above mixture was added to 100 *μ*L 95% ethanol and fully shocked 30 s. (3) The mixture in step 2 was added to 100 *μ*L chloroform and thoroughly mixed for 1 min, which was centrifuged at 3500 r/min for 8 min. The supernatant was SOD extract. (4) Next, detection of SOD was measured strictly in accordance with the procedure for SOD test kit.

The detection steps of erythrocyte GSH-Px were as follows. (1) 20 *μ*L of erythrocyte sedimentation was added to 480 *μ*L deionized water and fully mixed for 5 min until the mixture shows a fully transparent state. (2) Next, detection of GSH-Px was measured strictly in accordance with the procedure for GSH-Px test kit.

The detection steps of erythrocyte MDA were as follows. (1) 20 *μ*L of erythrocyte sedimentation was added to 180 *μ*L 1× hypotonic solution (0.01 M Tris-HCl, PH = 7.4) and fully mixed, which was centrifuged at 12000 r/min for 10 min after keeping hemolysis at 4°C for 30 min. The above mixture was discarded the supernatant. The above procedure was repeated for four times. (2) Isolation of erythrocyte ghost membranes was added to 30 *μ*L PIPA lysis buffer which has been added to PMSF inhibitors. The mixture should be repeated pipetting and recracking which was incubated on ice for 10 min. (3) Next, protein concentration was determined by the bicinchoninic acid (BCA) method. (4) Detection of MDA was measured according to the procedure for MDA test kit.

The serum contents of SOD, GSH-Px, and MDA were detected according to the procedure for SOD, GSH-Px, and MDA test kit.

#### 2.6.4. RNA Isolation and RT-PCR

Total RNA was isolated from mononuclear cells using Trizol reagent according to manufacturer's instructions. The total RNA concentration was quantified, and total RNA (5 *μ*g) was reverse-transcribed to cDNA using an RT-PCR kit. Reverse transcription was performed at 42°C for 60 min followed by inactivation at 70°C for 5 min. The resulting cDNA was further used as a template for polychain reaction (PCR) amplification immediately or stored at −40°C until use. Primer pairs of genes were synthesized and the parameters are included in Table 1. Real-time PCR was performed according to the protocol of the Qiagen Sybr Green PCR Kit in the Optical 96-Well Reaction Plate produced by applied biosystems (batch number 8010560) and *β*-actin as the endogenous control. The PCR conditions were 95°C for 10 min, 95°C for 30 s, annealing for 35 s, 72°C for 50 s, repeating for 10 cycles, and 72°C for 8 min. The different annealing temperatures were *α*-, *β*-, ^A^ *γ*-, ^G^ *γ*-globin, 55°C; SPTA1, SPTB, EPB4.1, 55°C; DNMT1, DNMT3a, DNMT3b, 60°C; BCL11A 54°C. PCR products were analyzed using a 1.2% agarose gel. Relative gene expression was calculated using the comparative threshold cycle (2^−ΔΔCt^) method. The sequences of gene-specific primers are summarized in Table 1.

#### 2.6.5. Western Blot Analysis

Western blot analysis was used to determine DNMT1, DNMT3a, and DNMT3b content in the whole blood for *α*-thalassemia. Protein was extracted according to the manufacturer's protocol. The protein concentration was determined by the bicinchoninic acid (BCA) method and equal amounts loaded on a 10% sodium dodecyl sulfate- (SDS-) polyacrylamide gel for electrophoresis. Protein bands were then transferred onto polyvinylidene fluoride (PVDF) membranes, which were stained by Ponceau staining reagents after the completion of transfer membrane. The membrane was completely immersed in phosphate-buffered solution with Tween (TBST) containing 5% bovine serum albumin (BSA) for 1 h. The membranes were incubated with primary antibodies overnight at 4°C. The mouse monoclonal anti-DNMT1 antibody and anti-DNMT3a antibody were diluted 1 : 1,000. The rabbit monoclonal anti-DNMT3b antibody was diluted 1 : 1,1000. Following incubation, membranes were washed three times in PBS with 0.1% Tween-20 for 10 min once. After that, horseradish peroxidase-conjugated goat anti-mouse and rabbit antibody (diluted 1 : 1,10000 in 5%BSA-TBST) were applied to the membrane for 40 min. Then, membranes were washed three times in PBS with 0.1% Tween-20 for 10 min once. Reactive proteins were detected on film using a chemiluminescent solution obtained from Millipore, USA. And the bands were then quantified by densitometry using Gelpro 3.2 software.

#### 2.6.6. TEM Observation of Inclusion Bodies in Erythrocytes

The procedure of measurement for inclusion bodies in erythrocytes was referred to the method of Wang et al. [9].

#### 2.6.7. Detection of Na^+^K^+^, Ca^2+^Mg^2+^, and T-ATP Enzyme in Erythrocytes

ATP enzymes can break down ATP to generate ADP and inorganic phosphate which content can be used to determine the level of ATP. The detection steps of Na^+^K^+^, Ca^2+^Mg^2+^, and T-ATPtase were as follows: 50 *μ*L of erythrocyte sedimentation was added to 450 *μ*L deionized water and fully mixed until observing that it was transparent. Detection of Na^+^K^+^, Ca^2+^Mg^2+^, and T-ATPtase activity was measured strictly in accordance with the procedure for Mini ATP enzyme test kit.

### 2.7. Statistical Analysis

Statistical analysis was performed using SPSS17.0. The results were presented as mean ± standard deviation. Paired* t*-test was used in comparing pre- and posttreatment. A *P* < 0.05 was considered as having statistical significance.

### 2.8. Medical Ethics

This study was approved by Ethics Committees of Guang'anmen Hospital, China Academy of Chinese Medical Sciences. All patients have signed the informed consents before entering trials, comprehensively understanding the purpose, procedures, possible risks, and benefits on participating in this study.

## 3. Results

All the 16 patients completed the whole observation without dropout.

### 3.1. YSSXG Can Improve Levels of Blood Parameters of Patients with Thalassemia Disease

**Figure 1:**
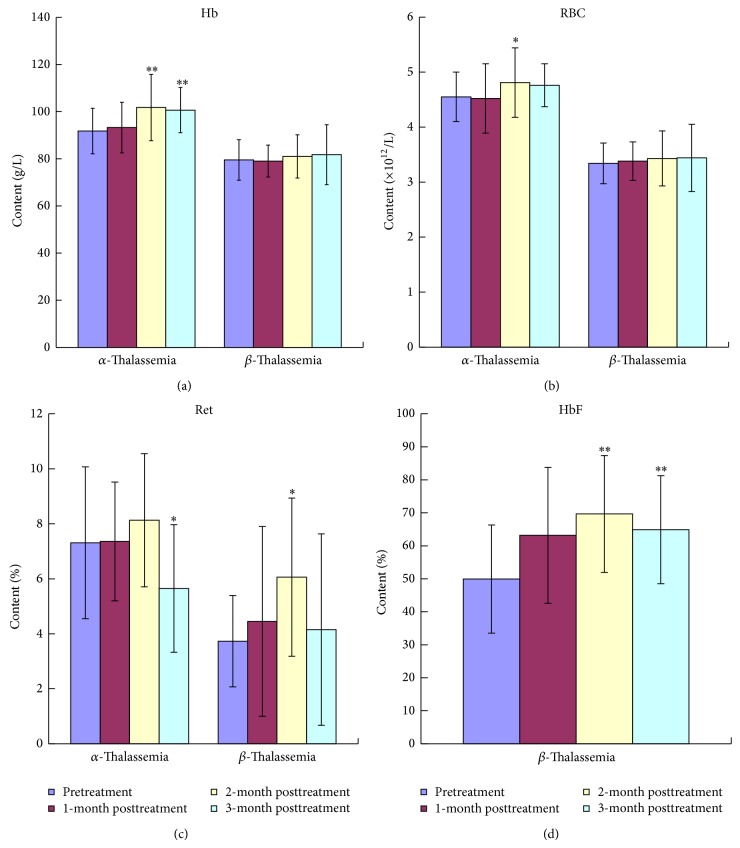
Effect of YSSXG on blood parameters of patients with *α*-thalassemia and *β*-thalassemia disease at pre- and posttreatment (*n* = 8, resp.). ^*^ *P* < 0.05, ^**^ *P* < 0.01, compared with pretreatment. (a) The changes of Hb concentration. (b) The changes of RBC counts. (c) The changes of Ret level. (d) The changes of HbF level of patients with *β*-thalassemia disease.

For *α*-thalassemia patients, levels of Hb concentrations and RBC counts from 1 to 3 months were higher than the levels of pretreatment. Levels of Hb at 2 and 3 months and RBC counts at 3 months were significantly increased compared with the measurement before treatment (*P* < 0.01 and *P* < 0.05, resp., Figures 1(a) and 1(b)). The Ret concentrations markedly decreased in the 3-month posttreatment (*P* < 0.05, Figure 1(c)).

For *β*-thalassemia patients, levels of Hb concentrations and RBC counts had kept on increasing in 3 months of treatment, while differences were not statistically significant compared with the measurements of pretreatment (Figures 1(a) and 1(b)). The measurement of Ret had an obvious increase at 2 months of posttreatment (*P* < 0.05, Figure 1(c)). Levels of HbF after treatment from 1 to 3 months were significantly increased compared with the measurements prior to treatment (*P* < 0.01 and *P* < 0.05, Figure 1(d)).

### 3.2. YSSXG Can Promote the Balance of Globin Chain Ratio of Patients with *α*-Thalassemia and *β*-Thalassemia Disease

For *α*-thalassemia patients, the relative expression of *α*-globin to *β*-globin was markedly increased compared with that of pretreatment (*P* < 0.01), and relative expressions of ^A^ *γ* and ^G^ *γ*-globin to *β*-globin had no statistically change compared with levels of pretreatment (Table 2).

For *β*-thalassemia patients, relative expressions of *β*-globin and ^G^ *γ*-globin to *α*-globin had no statistically change compared with that of pretreatment (*P* < 0.01), and the relative expressions of ^A^ *γ*-globin to *α*-globin had an increasing trend compared with levels of pretreatment (Table 3).

### 3.3. YSSXG Can Downregulate the mRNA Expression and Decrease the Activity of DNA Methyltransferase of Patients with *α*-Thalassemia Disease

**Figure 2:**
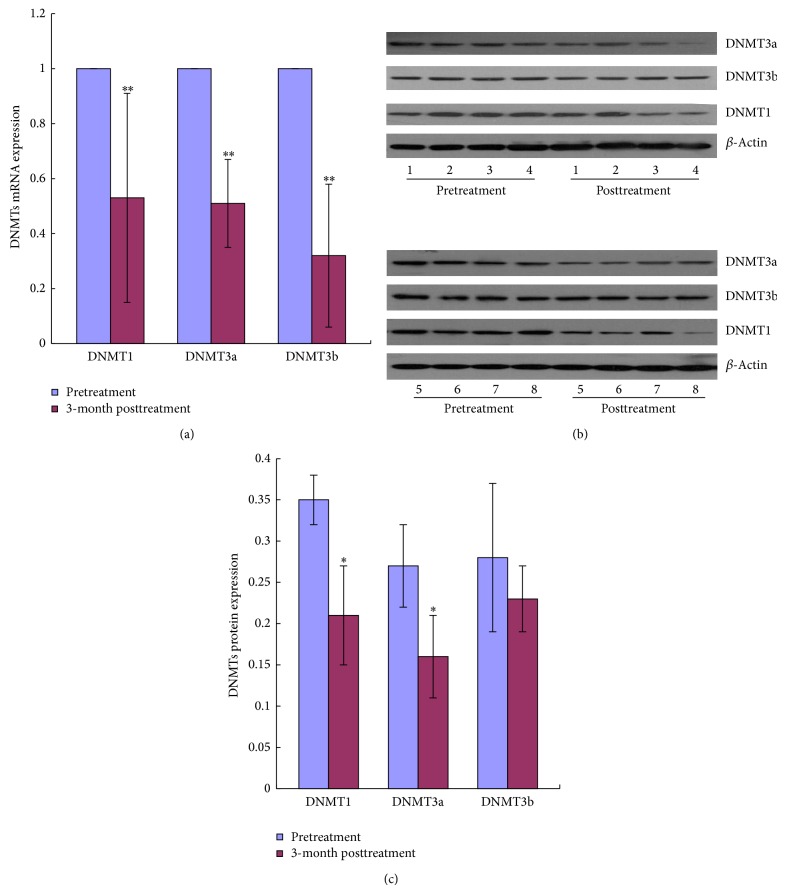
Effect of YSSXG on the mRNA expression and the protein activity of DNA methyltransferase of patients with *α*-thalassemia disease (*n* = 8). ^*^ *P* < 0.05, ^**^ *P* < 0.01, compared with pretreatment. (a) The fold changes relative of DNMTs mRNA expression level to pretreatment. (b) Electrophoresis of Western blot of DNMTs. Note that the number of 1 to 8 represents the sample of 8 patients with *α*-thalassemia disease, respectively. (c) The changes of protein expression level of DNMTs.

mRNA expressions of DNMT1, DNMT3a, and DNMT3b markedly decreased when compared with the level of pretreatment (*P* < 0.05, Figure 2(a)). Western blotting showed protein expression of DNMT1, DNMT3a, and DNMT3b (Figure 2(b)). The protein expression of DNMT1 and DNMT3a significantly decreased after the treatment of YSSXG (*P* < 0.01). The protein expression of DNMT3b had a decreasing trend (Figure 2(c)).

### 3.4. YSSXG Can Downregulate BCL11A mRNA Expression of Patients with *β*-Thalassemia Disease

**Figure 3:**
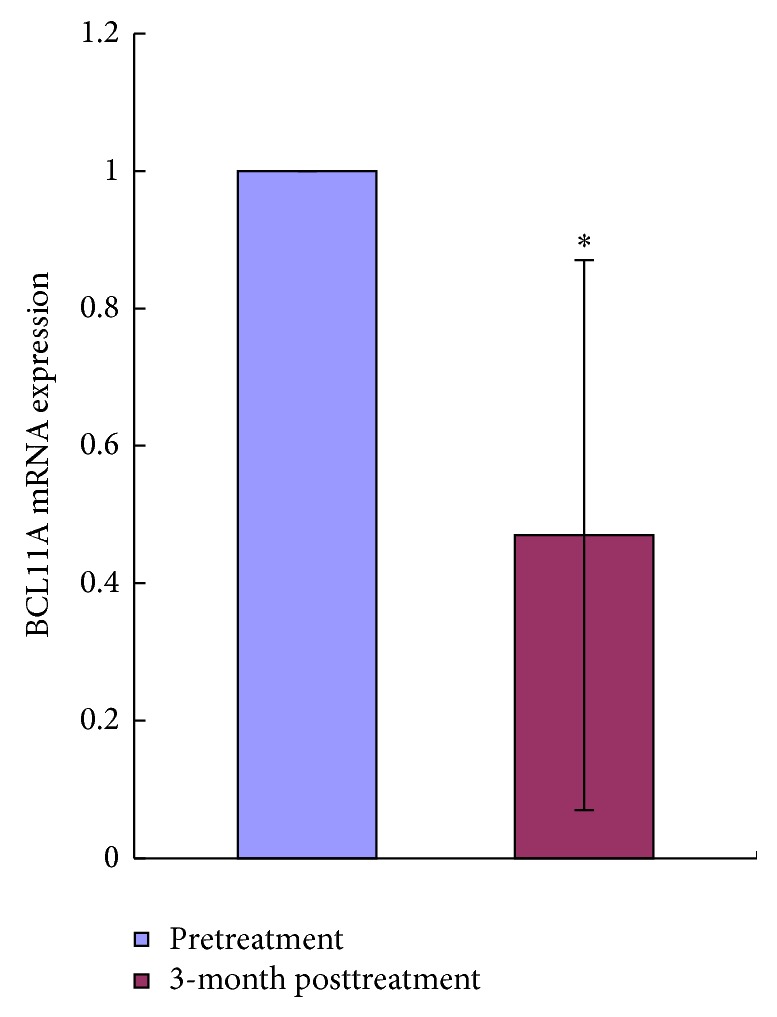
Effect of YSSXG on the mRNA expression of BCL11A of patients with *β*-thalassemia disease (*n* = 8). The fold changes relative of BCL11A mRNA expression level to pretreatment. ^*^ *P* < 0.05, compared with pretreatment.

The level of BCL11A expression of pretreatment was significantly higher than that of posttreatment (*P* < 0.05, Figure 3).

### 3.5. YSSXG Can Improve the Pro- and Antioxidative System Balance of Erythrocyte and Blood Serum of Patients with Thalassemia Disease

**Figure 4:**
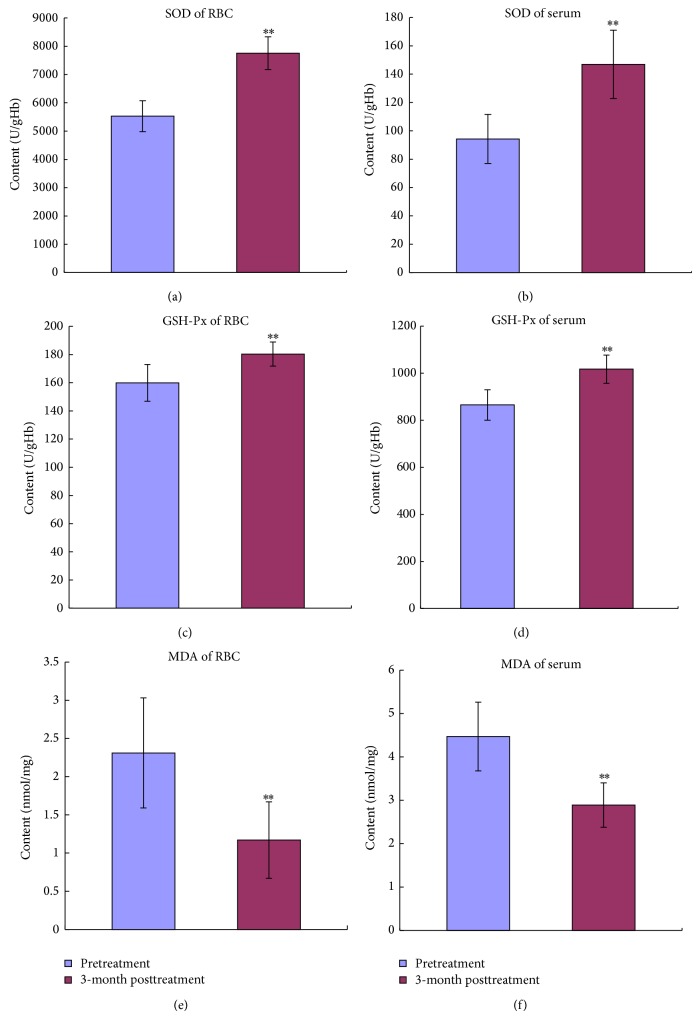
Effect of YSSXG on biomarkers of pro- and antioxidative system with thalassemia disease (*n* = 16). (a) The SOD activity level of RBC. (b) The SOD activity level of serum. (c) The GSH-Px activity level of RBC. (d) The GSH-Px activity level of serum. (e) The MDA activity level of RBC. (f) The MDA activity level of serum. ^*^ *P* < 0.05, ^**^ *P* < 0.01, compared with pretreatment.

After treatment with YSSXG, SOD and GSH-Px activities in erythrocytes and blood serum were increased significantly (*P* < 0.01, Figures 4(a), 4(b), 4(c), and 4(d)) and the MDA concentrations in RBCs and blood serum were obviously decreased (*P* < 0.01, Figures 4(e) and 4(f)).

### 3.6. YSSXG Can Upregulate the mRNA Expression of Erythrocyte Membrane Skeleton Protein of Patients with Thalassemia Disease

The mRNA expression of SPTA1 and SPTB of erythrocyte membrane skeleton protein markedly increased compared with levels of pretreatment (*P* < 0.05). The mRNA expression of EPB4.1 had an increasing trend, whereas the difference had no statistical change compared to the level of pretreatment (Table 4).

### 3.7. YSSXG Can Decrease the Content of Inclusion Bodies in Erythroid Cells of Patients with Thalassemia Disease

**Figure 5:**
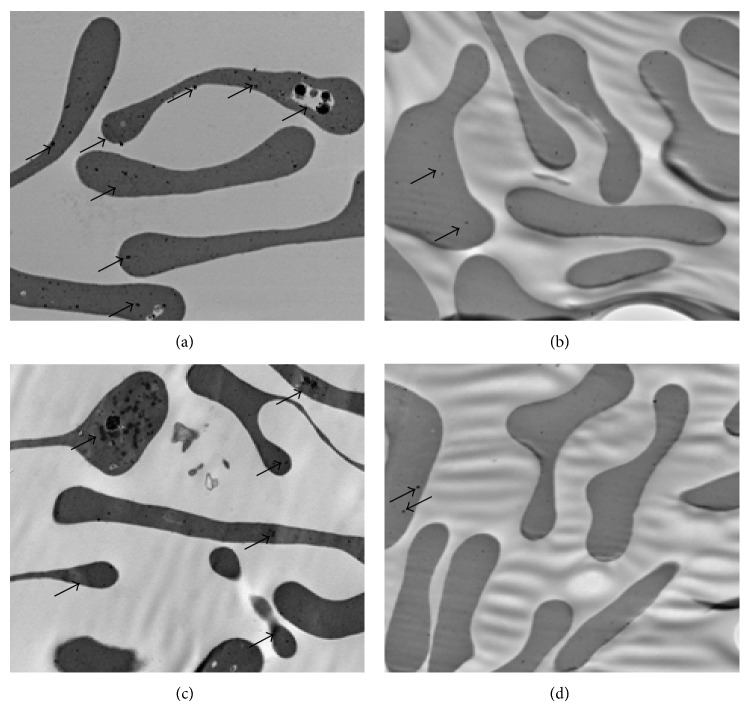
Effect of YSSXG on the mRNA expression of erythrocyte membrane skeleton protein of patients with thalassemia disease. (a) TEM images of inclusion bodies of *α*-thalassemia patients of pretreatment. (b) TEM images of inclusion bodies of *α*-thalassemia patients of posttreatment. (c) TEM images of inclusion bodies of *β*-thalassemia patients of pretreatment. (d) TEM images of inclusion bodies of *β*-thalassemia patients of posttreatment.

TEM images of erythroid cells are shown in Figure 5. RBCs had numerous dark grains indicative of inclusion bodies formed by unmatched denatured *β*-globin chains with *α*-thalassemia patients (Figure 5(a)), which were also observed on the *β*-thalassemia patients formed by unmatched denatured *α*-globin chains before treatment with YSSXG (Figure 5(c)). After treatment with YSSXG, the mount and volume of inclusion bodies decreased in two types of thalassemia (Figures 5(b) and 5(d)).

### 3.8. YSSXG May Increase Activities of Na^+^K^+^-ATPtase and T-ATPtase of Erythrocyte

**Figure 6:**
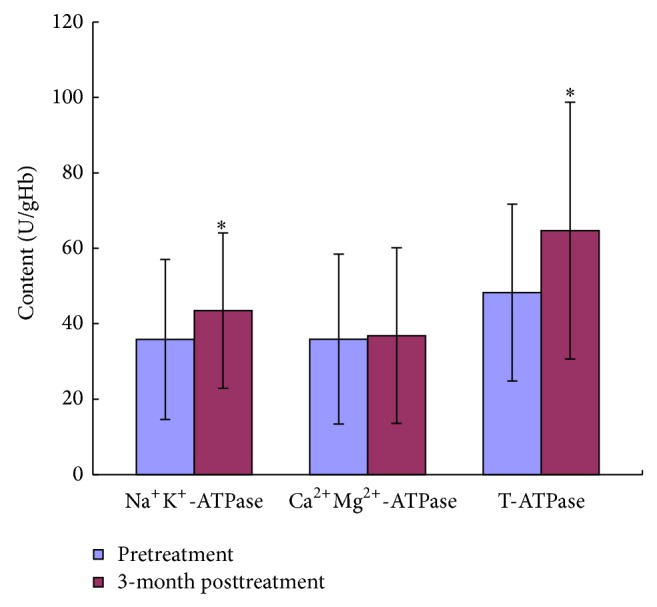
Effect of YSSXG on activities of Na^+^K^+^-ATPtase, Ca^2+^Mg^2+^-ATPtase, and T-ATPtase on erythrocyte of patients with thalassemia disease (*n* = 16). ^*^ *P* < 0.05, compared with pretreatment.

After treatment with YSSXG, Na^+^K^+^-ATPtase and T-ATPtase activities of Erythrocyte were increased significantly (*P* < 0.05) and Ca^2+^Mg^2+^-ATPtase activity had no significant change (Figure 6).

## 4. Discussion

Thalassemia belongs to “Blood Deficiency” or “Consumption” category in Chinese medicine. Based on the investigation of etiology, clinical manifestations, TCM syndromes, and genetic background, the professor of Wu Zhikui proposes that “congenital deficiency, kidney marrow damage, and blood metaplasia passive” are the core of the pathogenesis of thalassemia and then establishes the therapeutic principle which is “kidney-nourishing and marrow-replenishing.” The composition of Yisui Shengxue Granule is based on the traditional Chinese medicine theory of “kidney-nourishing and marrow-replenishing” and clinical practice, which is composed of 11 herbs complexes. The thalassemia syndrome is classified according to which of the globin chains, *α* or *β*, is affected. These 2 major groups, *α*- and *β*-thalassemia, are subclassified according to absent (*α*0 and *β*0) or reduced (*α*+ and *β*+) globin chain synthesis [10]. Difference in the amount of fetal hemoglobin (HbF) that persists into adulthood affects the severity of *β*-thalassemia syndromes [11]. *γ*-globin (a *β*-globin-like molecule), which binds to *α*-chains to produce HbF, addresses the imbalance in globin chains and this, in turn, reduces the occurrence of ineffective erythropoiesis, decreases hemolysis, and increases total Hb [12].

As observed in this experiment, the levels of Hb significantly increased and the Ret concentrations markedly decreased in the 3-month posttreatment for *α*-thalassemia patients, which indicated that the degree of anemia and ineffective hematopoiesis were markedly improved. Hb level did not increase significantly, but the level of HbF significantly elevated after 3-month treatment. Scores of symptoms were significantly lower than those before treatment, which showed that the improvement of clinical symptoms was consistent with levels of improvement in blood parameters. Hb level is one of the most important blood parameters in patients with thalassemia, which reflect the severity of thalassemia disease condition. By analyzing, we found that Hb levels of *β*-thalassemia patients were significantly lower than *α*-thalassemia patients, indicating that *β*-thalassemia patient's condition is generally more serious than *α*-thalassemia patients, which may be partly explained by the fact that the clinical efficacy of *α*-thalassemia was better than that of *α*-thalassemia patients by using Yisui Shengxue Granule, but it needs a large sample of clinical trials by further verification.

Human hemoglobin from embryonic (*α*2 *ε*2) is converted to the fetus (*α*2 *γ*2), and then from the fetus (*α*2 *γ*2) it is transmitted to adult (*α*2 *β*2), which are two different developmental stages. The *α*-globin genes, which are surrounded by widely expressed genes in a gene dense region of the genome, are silenced very early in development via recruitment of the Polycomb (PcG) complex [13]. The PcG complex seems to be recruited to the *α*-cluster by sequences within the CpG islands associated with their promoters [13]. The promoters of the human *α*-globin genes lie within large CpG islands. CpG methylation is thought to be carried out by different enzymes, the “denovo” MTases Dnmt3a and Dnmt3b and “maintenance” MTase Dnmt1, respectively [14]. DNA methylation plays an important role in transcriptional repression [14]. DNA methylase may affect the expression of *α*-globin by adjusting the level of DNA methylation in gene promoter region GpG islands. BCL11A gene regulates hemoglobin gene conversion and directly inhibits *γ*-globin gene transcription and then silences *γ*-globin gene [15–17]. The results of this study showed that for *α*-thalassemia patients, the relative expression of *α*-globin to *β*-globin markedly increased and levels of mRNA expression and protein expression decreased compared to levels of pretreatment, which indicated that the mechanism of clinical efficacy is partly attributed to improvement of globin chain ratio by inhibiting the expression of DNA methyltransferase. For *β*-thalassemia patients after YSSXG treatment, the relative expression of A *γ*-globin to *α*-globin had an increasing trend, and BCL11A expression level of posttreatment was significantly lower than that of pretreatment, which stated that the clinical efficacy for *β*-thalassemia patients partly accounted for increasing HbF level through reducing the expression of BCL11A. The increasing extent of HbF content was consistent with the decreasing extent of BCL11A expression.

The tetramer of normal adult hemoglobin is synthesized mainly by two *α*-globin chains and two *β*-globin chains (*α*2 *β*2). Human globin tetramer (*α*2 *β*2) in the body is stable, but free *α*-chain (*β*-thalassemia) or *β*-chain (*α*-thalassemia) in the body is unstable. The excess *α*-chain (*β*-thalassemia) or *β*-chain (*α*-thalassemia) form unstable homotetramers that precipitate on RBCs as inclusion bodies [13]. *α*-Homotetramers in *β*-thalassemia are more unstable than *β*-homotetramers in *α*-thalassemia and therefore precipitate earlier in the RBC life span, causing marked RBC damage and severe hemolysis associated with ineffective erythropoiesis and extramedullary hemolysis [18]. Iron overload, precipitated globin chains and premature hemolysis of red cell are contributing causes of oxidative stress in thalassemic patients [19–22]. The balance between the prooxidant and antioxidant levels becomes impaired while a decrease occurs in levels of antioxidant enzymes, an increase occurs in levels of MDA [23]. The degree of oxidative stress as indirectly measured by the alteration of antioxidant enzymes such as superoxide dismutase (SOD), glutathione peroxidase (GSH-Px) and catalase [24–26] or the products of lipid peroxidation such as malondialdehyde (MDA) content [27–29]. The precipitation of globin chain [30] and oxidative damage [31] induced by *α*- or *β*-globin chains which are associated with the membrane skeleton have been found to interact and disrupt the RBC membrane, damaging the cytoskeleton and resulting in differential membrane alterations. Na^+^K^+^-ATP enzyme and Ca^+^-Mg^+^-ATP enzyme on RBCs membrane can maintain a stable ion concentration and a normal morphology of RBC. Oxygen free radicals produced by lipid peroxidation inhibit the activity of erythrocyte membrane proteins which play a role in ion pump, such as Na^+^K^+^-ATP enzyme and Ca^2+^Mg^2+^-ATP enzyme, increase RBCs membrane permeability, and result in cell swelling and a decline in RBCs deformation [32].

TEM showed that RBCs of *α*-thalassemia and *β*-thalassemia patients were distributed in large number of dark re-dye particles before treatment, and then dark re-dye particles significantly reduced after treatment, which indicated that relative excess unpaired globin chains obviously reduced, suggesting that globin chain ratio tends to balance. The activity of SOD and GSH-Px of RBCs and serum, antioxidative damage indicators, significantly increased when compared with those of pretreatment. And the activity of MDA of RBCs and serum, oxidative damage indicator, significantly decreased when compared with that of pretreatment. Those suggest that the improvement of pro- and antioxidative system balance is directly related to the alleviation of hemolysis and anemia by Yisui Shengxue Granule treatment. The mRNA expressions of SPTA1, SPTB, and EPB4.1, mainly erythrocyte membrane skeleton protein, were markedly increased after treatment, and the activities of Na^+^K^+^-ATPtase and T-ATPtase of erythrocyte were also significantly increased, which are the causes of Yisui Shengxue Granule promoting the intact of erythrocyte morphology and the recovery of erythrocyte function.

## 5. Conclusion

Yisui Shengxue Granule to treat thalassemia disease has an affirmative clinical efficacy. Mechanisms of YSSXG improving hemolysis and anemia of erythrocytes are as follows: promoting a balanced ratio of globin chains, inhibiting DNA methyltransferase activity and BCL11A mRNA expression, improving antioxidant ability of erythrocyte, reducing inclusion content of erythrocyte, and improving the structure and function of erythrocyte.

## Figures and Tables

**Table 1:** Primer used for detecting the gene expression by reverse real-time PCR.

Target gene	Primer sequence 5′-3′		Predicted amplification segments (bp)
	Forward primer	Reverse primer	
*β*-Actin	GAG ACC TTC AAC ACC CCA GCC	AAT GTC ACG CAC GAT TTC CC	263
SPTA1	GAT CTT GAA GCC AAT GTC CA	CAA CTC CCT CCA CTG GTG	250
SPTB	AAC CGG GAT AAG GTC TTG AGT C	GGT GCT TTA GCC ATT TAT TGT GA	289
EPB4.1	TTA TCC ACT CAC TCA CCC TTC C	CTC ATC AGC AAT CTC GGT CTC C	369
BCL11A	CGC CAG AGG ATG ACG AT	CAG GCG TGG GGA TTA GA	138
*α*-Globin	CCC ACC ACC AAG ACC TAC TT	GCT CAC AGA AGC CAG GAA CTT	291
*β*-Globin	TAG CAA CCT CAA ACA GAC ACC A	ATC ACT AAA GGC ACC GAC CAC T	243
^A^ *γ*-Globin	ATC AAT AAG CTC CTA GTC CAG ACG	CCA AAG CTG TCA AAG AAC CTC T	166
^G^ *γ*-Globin	AGG AGG ACA AGG CTA CTA TCA CAA	CTT GAG ATC ATC CAG GTG CTT TAT	233
DNMT1	TAC CTG GAC GAC CCT GAC CTC	CGT TGG CAT CAA AGA TGG ACA	103
DNMT3a	TAT TGA TGA GCG CAC AAG AGA GC	GGG TGT TCC AGG GTA ACA TTG AG	111
DNMT3b	GGC AAG TTC TCC GAG GTC TCT G	TGG TAC ATG GCT TTT CGA TAG GA	132

**Table 2:** Effect of YSSXG on globin chain ratio of *α*-thalassemia patients pre- and posttreatment ($\overset{-}{x}\pm s$).

Therapy time	Case	2^−Δct (α)^/2^−Δct (*β*)^	2^−Δct (A_*γ*_)^/2^−Δct (*β*)^	2^−Δct (G_*γ*_)^/2^−Δct (*β*)^
Pretreatment	8	0.80 ± 0.62	0.13 ± 0.09	0.14 ± 0.17
3 month posttreatment	8	2.02 ± 0.98	1.02 ± 1.33	0.33 ± 0.35
3 month posttreatment versus pretreatment (95%CI)/*P* value		0.004^##^	0.097	0.168

Notes. ^#^ *P* < 0.05, ^##^ *P* < 0.01.

**Table 3:** Effect of YSSXG on globin chain ratio of *β*-thalassemia patients pre- and posttreatment ($\overset{-}{x}\pm s$).

Therapy time	Case	2^−Δct (*β*)^/2^−Δct (*α*)^	2^−Δct (A_*γ*_)^/2^−Δct (*α*)^	2^−Δct (G_*γ*_)^/2^−Δct (*α*)^
Pretreatment	8	0.07 ± 0.06	0.46 ± 0.18	0.62 ± 0.55
3 month posttreatment	8	0.07 ± 0.06	1.27 ± 1.54	0.59 ± 0.39
3 month posttreatment versus pretreatment (95%CI)/*P* value		0.956	0.165	0.864

Notes. ^#^ *P* < 0.05, ^##^ *P* < 0.01.

**Table 4:** Effect of YSSXG on erythrocyte membrane skeleton protein gene expression pre- and posttreatment ($\overset{-}{x}\pm s$).

Therapy time	Case	SPTA1	SPTB	EPB4.1
Pretreatment	16	0.33 ± 0.40	0.12 ± 0.16	0.32 ± 0.33
3 month posttreatment	16	1.17 ± 1.50	0.41 ± 0.43	0.87 ± 1.34
3 month posttreatment versus pretreatment (95%CI)/*P* value		0.046^#^	0.018^#^	0.069

Notes. ^#^ *P* < 0.05, ^##^ *P* < 0.01.
